# Epidemiology and contemporary risk profile of traumatic spinal cord injury in Switzerland

**DOI:** 10.1186/s40621-015-0061-4

**Published:** 2015-11-02

**Authors:** Jonviea D. Chamberlain, Olivier Deriaz, Margret Hund-Georgiadis, Sonja Meier, Anke Scheel-Sailer, Martin Schubert, Gerold Stucki, Martin WG Brinkhof

**Affiliations:** 1 Swiss Paraplegic Research, Guido A. Zäch Strasse 4, CH-6207 Nottwil, Switzerland; 2 Department of Health Sciences and Health Policy, University of Lucerne, Lucerne, Switzerland; 3 Clinique Romande de Réadaption (CCR) and Institut de recherche en réadaptation, Sion, Switzerland; 4 REHAB Basel, Basel, Switzerland; 5 Swiss Paraplegic Center, Nottwil, Switzerland; 6 Spinal Cord Injury Center, Balgrist University Hospital, Zurich, Switzerland

**Keywords:** Epidemiology, Spinal cord injury, Incidence, Chronic, Injury

## Abstract

**Background:**

Traumatic spinal cord injury (TSCI) has a high personal and socio-economic impact. Effective public health prevention policies that aim to reduce this burden are reliant on contemporary information of the risk and underlying causes of TSCI. This study contextualizes Swiss annual incidence rates within the European context, and provides detailed estimates by age, gender and etiology towards informing targeted intervention strategies.

**Methods:**

TSCI cases that occurred in the years 2005 to 2012 were identified as part of the Swiss Spinal Cord Injury (SwiSCI) cohort study through a rehabilitation-based study of local medical files.

**Results:**

The crude annual incidence rate (IR) estimate of TSCI for the study period was 18.0 (95 % confidence interval 16.9–19.2) per one million population; standardized to the WHO world population IR was 21.7 (20.3–23.1) population. The injury rate of TSCI in Switzerland was intermediate in comparison to estimates for other European countries, which ranged from around 8.3 in Denmark to 33.6 per million in Greece. Males exhibited consistently higher IRs than females, with a highest IR ratio (IRR) of 3.9 (2.8–5.5) in young adults (aged 16 to 30). Sports and leisure and transport-related injuries were the predominant causes of TSCI in the youngest age group (aged 16 to 30); falls were the predominant cause among the oldest age group (76 years or over). With increasing age, a greater proportion of fall-related TSCIs were due to low-level falls, with more than 80 % of fall-related TSCIs due to low-level falls in the oldest age group.

**Conclusions:**

Evidence suggests sports/leisure- and transport-related injuries in young men and falls among the elderly as prime targets for prevention policies and programs.

## Background

Traumatic spinal cord injuries (TSCIs) are a rare, albeit oftentimes life-altering condition with long-term physical, psychological, social and financial implications (WHO 2013; Branco et al. 2007; Post and van Leeuwen 2012). TSCIs can result in lasting neurologic impairments of all organ systems and body functions below the neurologic lesion level, thereby causing the loss of function, decreased mobility, increased morbidity and reduced life expectancy and quality-of-life. Related to the financial implications of TSCI, one study estimated the lifetime costs for a person injured at 25 years to be 4.6 million US$ for high tetraplegia, and 2.3 million for paraplegia (WHO 2013). To inform targeted public health interventions, policies, and resource management efforts aimed at prevention and improvement in the lives of individuals with TSCI, valid and reliable data on the basic epidemiological characteristics of TSCI (i.e., demographic characteristics, incidence and prevalence) are imperative (Ivers 2012).

Existing literature demonstrates large variations in TSCI incidence and etiology globally. Cultural and regional contexts are likely to influence country-level differences, as evidenced by cause-specific estimates of TSCI incidence, underscoring the necessity of country-specific estimates for mitigating the burden of TSCI (Devivo 2012; Lee et al. 2014; Singh et al. 2014; Jazayeri et al. 2014; WHO 2013). However, few contemporary estimates exist. Previous research has also demonstrated the importance of age and sex on incidence and etiology of TSCI, for example the increase in risk for falls with increasing age. Therefore, the worldwide aging of populations observed in many developed nations including Europe, TSCI is a growing health concern, necessitating contemporary estimates (Eurostat 2014).

The purpose of this study is to establish prevention priorities in Switzerland for policy makers, by estimating reliable, contemporary incidence rates of TSCI according to key demographic characteristics. Two publications currently exist for Switzerland that attempt to address the knowledge gap regarding the epidemiology of TSCI in Switzerland, although methodological and quantitative issues subsist, and given changing demographics (e.g., average age, and gender distribution) the estimates are also outdated (Eberhard 2004; Gehrig and Michaelis 1968). Consequently, no reliable, contemporary estimates for Switzerland exist. The Swiss Spinal Cord Injury (SwiSCI) Cohort Study serves as an ideal platform for estimating the burden of TSCI and identifying areas for prevention purposes, with results generalizable to other epidemiologically similar regions as data coverage is representative of the entire population, and is available by demographic and characteristics specific to spinal cord injuries (SCIs). This investigation aimed to describe etiology, demographic and SCI characteristics of patients receiving first rehabilitation in Switzerland and to calculate annual age- and sex-specific incidence rates of TSCI in Switzerland by demographic and injury characteristics using contemporary data collected in the SwiSCI study (Kirshblum et al. 2011; Post et al. 2011).

## Methods

### Coverage

**Fig. 1:**
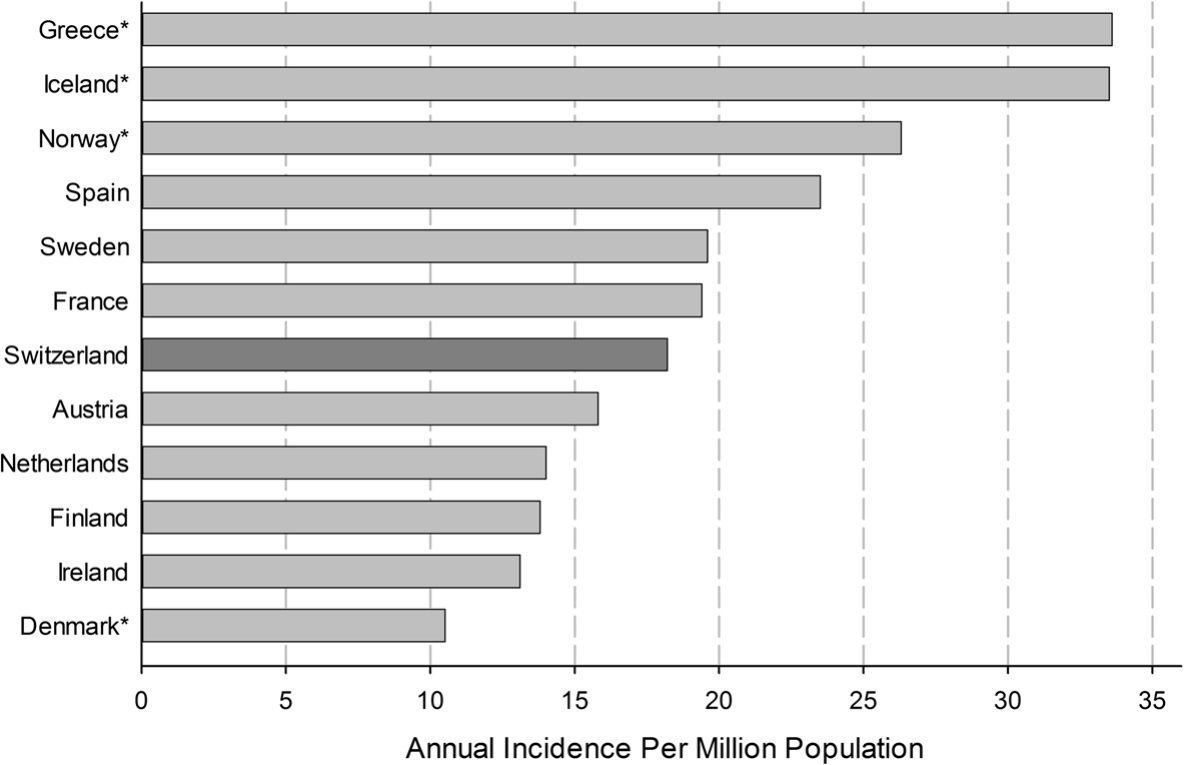
European estimates of annual TSCI incidence per million population. Comparison of reported annual incidence rates per million population for European countries with similar methodology. ^*^ Indicates estimates based on sub-national survey data. Country references: Greece (Divanoglou and Levi 2009); Iceland (Knutsdottir et al. 2012); Norway (Hagen et al. 2010); Spain (Perez et al. 2012); Sweden (Divanoglou and Levi 2009); France (Albert and Ravaud 2005); Austria (Jazayeri et al. 2014); Netherlands (Nijendijk et al. 2014); Finland (Ahoniemi et al. 2008); Ireland (O’Connor and Murray 2006); Denmark (Bjornshave Noe et al. 2015)

SwiSCI is a longitudinal, rehabilitation-based cohort study with the goal of understanding how to better support “functioning, health maintenance, and quality-of-life of persons with SCI” (Post et al. 2011). This study used data from a retrospective study of medical files from all four specialized rehabilitation centers for SCI in Switzerland (Post et al. 2011). The medical record study as described by Post et al. (Post et al. 2011) for 2005 to 2009 was extended until mid-2013. The extended study collects similar information to the originally planned study on demographic and lesion characteristics, but with fewer variables related to, for example, diagnosis or acute care. This study used data for the years 2005 until, and including, 2012. Data for the comparison chart (Fig. 1) were extracted from literature identified in recent systematic reviews, updated with recent literature not included in the systematic reviews, including only those countries with contemporary estimates (i.e., including data from 2000 or later), and from epidemiologically similar countries (e.g., with respect to income, etiological distribution of TSCI, geographic region) (WHO 2013; Lee et al. 2014; Jazayeri et al. 2014; Singh et al. 2014).

### Included cases

The present study reports on all diagnoses of acquired traumatic SCI that were recorded among persons aged 16 years and older, who started first rehabilitation in one of the four Swiss referral centers for SCI from January 1, 2005 to December 31, 2012 (Post et al. 2011). Only individuals that survived to be admitted to a rehabilitation center were thus included. Traumatic SCI was defined as the event of an acute traumatic lesion of neural elements in the spinal canal (spinal cord and cauda equina) that resulted in temporary or permanent sensory and/or motor deficit.

### Data management and access

All medical record data are stored on protected data servers at Swiss Paraplegic Research in Nottwil, controlled and administered by the SwiSCI Study Center data manager. Personal data extracted from medical records, stored to enable individual follow-up or future linkage to other data sources, are stored separately from the research data using a unique identifier (SwiSCI ID number). Potentially eligible individuals were first identified electronically at each specialized center, the medical records were then pulled and screened to verify study eligibility before manual extraction of data. Following the completion of the medical records study, a data check was completed to ensure that included cases met the eligibility criteria, and to remove duplicates by identifying records with identical demographic (name, address, birthday) and SCI (date of SCI; lesion level and severity). Additionally, a medical professional specialized in SCI rehabilitation systematically cross-checked clinical consistency of coding related to neurological classification of injury level and severity (American Spinal Injury Association (ASIA) Impairment Scale (AIS)) across different time periods during first rehabilitation by reviewing clinical assessment sheets showing substantial variation between level of motor and sensory impairment (i.e., two or more) (Lee et al. 2014; Kirshblum et al. 2011).

Use of data collected in the medical records study requires a formal research proposal submitted to the SwiSCI Study Center, following which two expert reviewers appraise and support the proposal before submission for a final decision by the SwiSCI Steering Committee. If approved, access to the data is governed by the data manager who anonymizes data to ensure subjects’ privacy. This study was approved by the principal ethics committee on research involving humans of the Canton Lucerne and endorsed by cantonal ethics committees (reference numbers: 1008 [Luzern]; 37/11 [Basel]; CCVEM 015/11 [Valais]; 2012–0049 [Zürich]).

### Analysis

Descriptive analyses of TSCI are reported stratified by key demographic characteristics and SCI-specific characteristics (e.g., sex, age at injury, ASIA Impairment Scale (AIS) at discharge, SCI type, and etiology), according to guidelines of the International Spinal Cord Society (ISCoS) where possible (e.g., not possible to follow guidelines in relation to lesion severity and level groupings for all cases) (DeVivo et al. 2011). The ASIA impairment scale (AIS) is an internationally recognized, clinician-administered standard, essential for the classification of neurological status, and which categorizes motor and sensory impairments by identifying the most superior spinal level demonstrating ‘unimpaired’ function. The ASIA impairment scale (AIS) classifies individuals on a five point scale from A (complete SCI) to E (normal sensory and motor function) (Kirshblum et al. 2011). Lesion levels were dichotomized into tetraplegia and paraplegia; tetraplegia referring to an impairment in the cervical spine region, and paraplegia referring to lesions within the thoracic, lumbar, or sacral spine regions (Kirshblum et al. 2011). Variables were grouped in this way due to the established impact on functioning, morbidity and mortality (WHO 2013). Categorization of falls into low (less than one meter) versus high falls (at least one meter) was based on available information on cause of injury in the medical records. A one meter cut-off for classifying level of falls was chosen to facilitate comparison with previous reports in the literature (Knutsdottir et al. 2012; Feng et al. 2011; Chen et al. 2015).

Annual incidence rates (IRs) were calculated using both the Swiss population as well as the WHO World Population, as recommended for providing globally comparable rates (Ahmad et al. 2000). Swiss population-based data, stratified by age and sex, were used for the denominator to calculate annual incidence rates per one million persons. Swiss population data were retrieved from the Swiss Federal Office of Statistics (Neuchâtel). Patient characteristics were stratified according to ISCoS guidelines (DeVivo et al. 2011). Age- and sex-specific annual incidence rates and their 95 % confidence intervals were calculated using Poisson distribution. Incidence rate ratios (IRRs) were calculated using a Poisson regression model, whereby the number of incident cases per year were used as count data (dependent variable), population counts were used as exposure data (denominator), and age, sex, and lesion type served as predictors (independent variable) for the incidence rate. Direct standardization was used to calculate annual age- and sex-adjusted incidence rates according to the WHO World population, provided to aid in future comparisons with other settings.

All analyses were conducted using STATA Version 13.1 for Windows (College Station, TX).

## Results

### Description of the study sample

From 2005 to 2012, there were 932 cases of traumatic SCI, of which 239 were female, and 693 were male. Paraplegia (*n* = 524; 56.2 %) was more common than tetraplegia. The average age for the study population was 48.0 (SD = 19.7) years. The age distribution is presented in Table 1. The largest number of new cases occurred in the age group of 16 to 30 years, the majority of which resulted in paraplegia. About 40 % of TSCI cases resulted in ASIA impairment scale (AIS) D (*N* = 370), with an additional 30 % of lesions between T1-S5 with an AIS A, B, or C; high, severe lesions only accounted for 17 % of cases (C1-C8; AIS A, B, or C) (Table 1). Persons incurring paraplegia were slightly younger than those with tetraplegia (mean age 43.8 and 53.5, respectively) (Table 1). A large proportion of the study population had ASIA impairment scale (AIS) D lesion severity (39.7 %). More cases of incomplete lesions than complete lesions were observed (68.1 and 28.3 %, respectively), with roughly similar distributions in males and females. Statistically significant differences were observed in SCI type according to gender, with a larger proportion of females experiencing paraplegia compared to males (65.3 and 53.1 %, respectively), while a higher proportion of males experienced tetraplegia in comparison with females (46.9 % and 34.7 %, respectively) (Pearson chi-square = 10.69; *p* = 0.001).

**Table 1:** Study characteristics by SCI type and overall

Variable	Tetraplegia	Paraplegia	Overall	*P*-value
Sex (n; %)				<0.001
Male	325 (79.7)	368 (70.2)	693 (74.4)	
Female	83 (20.3)	156 (29.8)	239 (25.6)	
Mean age at injury (Median; S.D.; Min-Max)	53.5 (56; 19.8; 16–93)	43.8 (40; 18.6; 16–90)	48.0 (47; 19.7; 16–93)	
Age at injury (n, %)				<0.001
16–30	72 (17.7)	158 (30.2)	230 (24.7)	
31–45	70 (17.2)	149 (28.4)	219 (23.5)	
46–60	94 (23.0)	110 (21.0)	204 (21.9)	
61–75	108 (26.5)	70 (13.4)	178 (19.1)	
76+	64 (15.7)	37 (7.1)	101 (10.8)	
Etiology (n; %)				<0.001
Sports and leisure	121 (29.7)	126 (24.1)	247 (26.5)	
Transport	103 (25.3)	110 (21.0)	213 (22.9)	
Fall	157 (38.5)	189 (36.1)	346 (37.1)	
Other cause	27 (6.6)	99 (18.9)	126 (13.5)	
AIS (n; %)				<0.001
A	81 (19.9)	183 (34.9)	264 (28.3)	
B	51 (12.5)	47 (9.0)	98 (10.5)	
C	83 (20.3)	84 (16.0)	167 (17.9)	
D	178 (43.6)	192 (36.6)	370 (39.7)	
E	7 (1.7)	5 (1.0)	12 (1.3)	
Unknown	8 (2.0)	13 (2.5)	21 (2.3)	
Lesion level and AIS (n; %)				
C1-C4; A,B,C	-	-	86 (9.2)	
C5-C8; A,B,C	-	-	71 (7.6)	
T1-S5; A,B,C	-	-	284 (30.5)	
All D	-	-	370 (39.7)	
All E	-	-	12 (1.3)	
Unknown	-	-	109 (11.7)	

*SD* standard deviation, *AIS* American Spinal Injury Association impairment scale, *P*-value from Pearson’s chi-square test comparing paraplegia and tetraplegia

### Overall incidence

The overall annual incidence rate between 2005 and 2012 was 18.0 per one million (95 % CI 16.9–19.2) (Table 2). Adjustment to the WHO world population resulted in similar, albeit slightly higher, overall annual incidence rates. In comparison with reported annual incidence rates by other nationally representative studies, overall incidence of TSCI for Switzerland was intermediately located (Fig. 1). Similar to previous literature, incidence of TSCI in males was consistently higher than that observed among females. The overall annual incidence rate was 27.5 (25.5–29.6) among men and 9.0 (7.9-10.2) for women, yielding an overall incidence rate ratio of 3.1 (2.7–3.6) (Table 2). The highest IRR of males compared to females was observed between the ages of 16 and 30 years (IRR = 3.9; 95 % CI 2.8–5.5), and the lowest between the ages of 46 and 60 years (2.7; 2.0–3.7) (Table 2). Although the largest proportion of cases were observed among individuals aged 16 to 30 years, the highest estimated annual IR was among individuals 76 and older (22.4; CI = 18.5–27.3). Annual incidence rates in males showed a convex relationship with age, with high IRs among men aged 16 to 30 years (31.6; 27.4–36.6) and men aged 76 and older (38.3; 30.0–49.0), while men aged 46–60 (22.9; 19.5–26.9) showed the lowest IR (Table 2).

**Table 2:** Annual age- and sex-specific incidence rates per one million population for TSCI and corresponding incidence rate ratios

	Incidence Rate (95 % CI)									
	Male			Female			Overall			Male to Female (IRR)
	Para	Tetra	*Total*	Para	Tetra	*Total*	Para	Tetra	*Total*	*Total*
16–30	20.8 (17.4–24.9)	10.8 (8.5–13.9)	31.6 (27.4–36.6)	6.5 (4.7–9.0)	1.6 (0.8–3.0)	8.1 (6.1–10.8)	13.8 (11.8–16.1)	6.3 (5.0–7.9)	20.1 (17.6–22.8)	3.9 (2.8–5.5)
31–45	15.4 (12.8–18.6)	8.5 (6.6–10.9)	23.9 (20.5–27.7)	5.7 (4.2–7.8)	1.4 (0.3–2.6)	7.1 (5.4–9.4)	10.6 (9.0–12.4)	5.0 (3.9–6.3)	15.6 (13.6–17.7)	3.4 (2.4–4.7)
46–60	11.8 (9.5–14.8)	11.1 (8.8–13.9)	22.9 (19.5–26.9)	5.1 (3.7–7.2)	3.4 (2.3–5.2)	8.5 (6.6–11.2)	8.5 (7.1–10.3)	7.3 (5.9–8.9)	15.8 (13.8–18.1)	2.7 (2.0–3.7)
61–75	9.9 (7.3–13.5)	20.8 (16.8–25.7)	30.7 (25.8–36.6)	6.4 (4.4–9.2)	4.8 (3.2–7.3)	11.2 (8.5–14.7)	8.1 (6.4–10.2)	12.4 (10.3–15.0)	20.5 (17.7–23.7)	2.7 (2.0–3.9)
76+	12.0 (7.7–18.6)	26.3 (19.6–35.4)	38.3 (30.0–49.0)	6.0 (3.7–9.7)	7.1 (4.6–11.0)	13.1 (9.5–18.1)	8.2 (6.0–11.4)	14.2 (11.2–18.2)	22.4 (18.5–27.3)	2.9 (1.9–4.5)
*Total*	12.9 (11.6–14.4)	14.6 (13.2–16.2)	27.5 (25.5–29.6)	5.9 (5.0–6.9)	3.1 (2.5–3.9)	9.0 (7.9–10.2)	10.1 (9.3–11.0)	7.9 (7.2–8.7)	18.0 (16.9–19.2)	3.1 (2.7–3.6)
Standardized to WHO Standard World Population										
*Total* ^*a*^	17.6 (15.9–19.5)	15.6 (14.0–17.4)	33.2 (30.8–35.7)	7.0 (6.0–8.2)	3.7 (3.0–4.6)	10.7 (9.5–12.2)	12.2 (11.2–13.3)	9.5 (8.6–10.4)	21.7 (20.3–23.1)	3.1 (2.7–3.6)

^**a**^Age-standardized to the WHO standard world population (Ahmad et al. 2000); IRR = Incidence Rate Ratio

Annual incidence rates for paraplegia were higher than for tetraplegia, until after the age of 61 years, among men, and greater than 76 years among women, when IRs of tetraplegia became higher. This difference was only observed among males, not females. With increasing age, the IRR of males compared to females among paraplegia decreased; no clear pattern was observed for tetraplegia.

### Cause-specific incidence rates

**Fig. 2:**
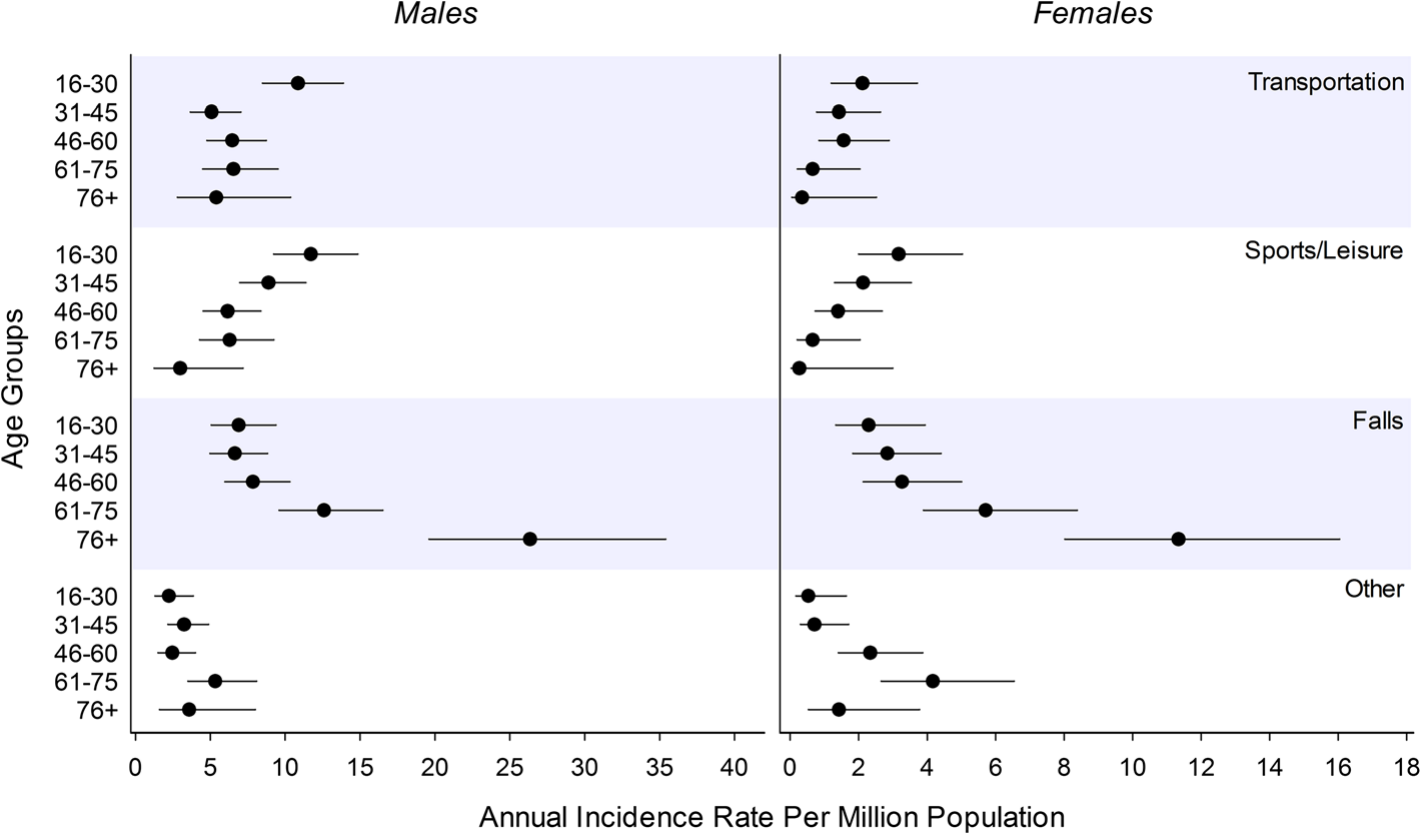
Annual incidence rates for males and females by cause of TSCI and age groups. Annual incidence rates of TSCI by etiology, according to gender and age group. Note that the IR per million population scale (x-axis) for men is different than that for females

**Fig. 3:**
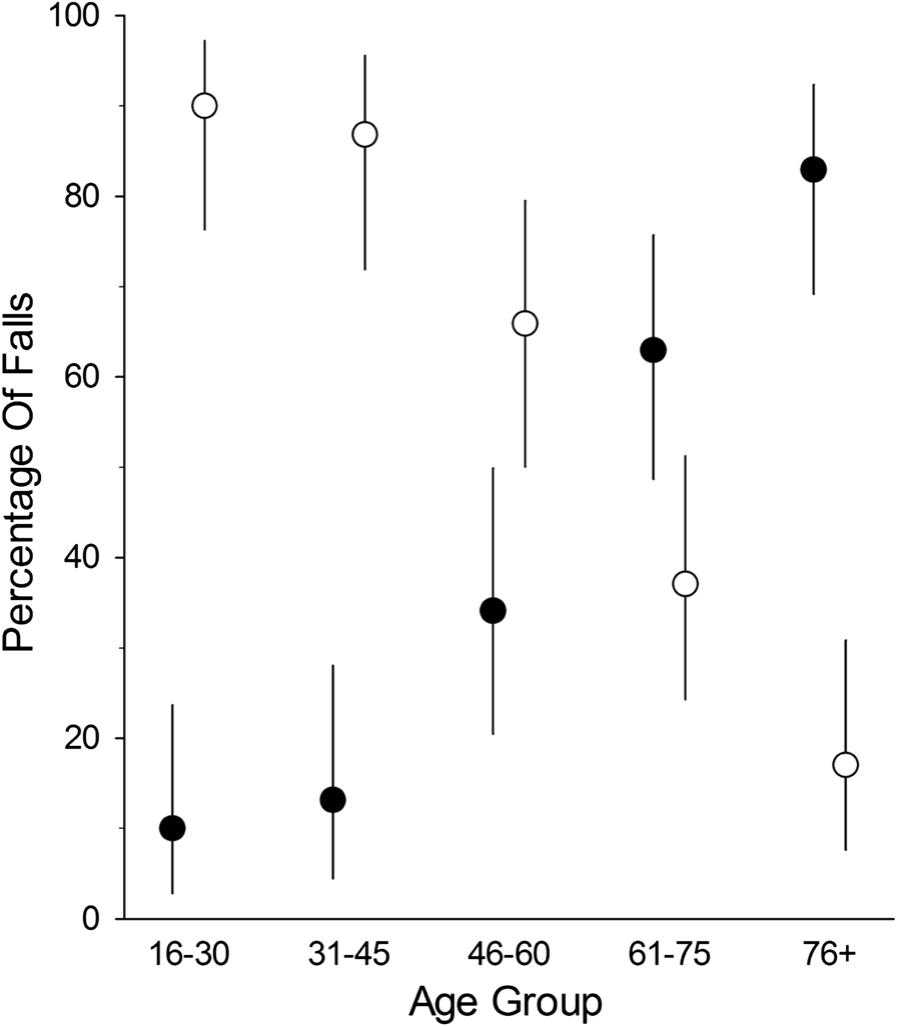
Percentages of high and low falls by age group. Percentages of fall-related TSCIs according to height of fall by age group. The filled-in, black circles indicate low-level falls (i.e., < 1 m); the unfilled, white circle indicates high-level falls (i.e., > 1 m). The vertical lines that dissect each circle represent the 95 % confidence intervals. Height of fall not known for 123 fall-related cases of TSCI (35.5 %)

The most common causes of TSCI were falls, sports/leisure-related activities, and transport-related injuries. Fall-related injuries accounted for 37.1 % (*N* = 346) of TSCI cases, with slightly more cases of paraplegias than tetraplegia (54.6 % versus 45.4 %, respectively) (Table 1). Significant differences were observed in the gender distribution by etiology, however, falls and sports/leisure-related injuries accounted for the leading cause of TSCI for both males and females. The variation in distribution and trends by age group and sex according to etiology signify the influence of age and sex on etiology (Fig. 2). For example, among females the annual incidence rates for falls increased with increasing age, with incidence rates of 2.3 (1.3–3.9), 2.8 (1.8–4.4), 3.3 (2.1–5.0), 5.7 (3.9–8.4), and 11.3 (8.0–16.0) per million population for 16–30, 31–45, 46–60, and 76 and older years of age, respectively; a similar trend was observed for males (Fig. 2). Conversely, the incidence of sport- and leisure-related injuries declined with increasing age in both men and women (Fig. 2). Transport-related TSCIs were largely attributed to car crashes (28.6 %), and motorcycle (28.6 %) and bicycle (13.1 %) crashes (Table 3). Other causes of transport-related TSCIs included, e.g., tractor accidents, a bus crash in Turkey, and non-specified incidents on public highways. Within the study population, specific causes of sports- and leisure-related TSCI could be attributed to skiing/snowboarding (21.4 %), paragliding/parachuting (19.8 %), and swimming/diving (14.5 %) (Table 3). Remaining causes included, for instance, hiking, horseback riding, mountain biking or bicycling, and motocross (Table 3). Among known causes, the majority of fall-related TSCIs were due to falling from a tree or off a ladder. Fall-related TSCIs appeared often age-dependent especially for TSCIs due to tripping (9.8 %) or tripping down stairs (11.6 %), given the observed increase in the proportion of TSCIs due to falls with increasing age (Table 3). Furthermore, as shown in Fig. 3, an age effect on level of falls was observed with the proportion of low-level falls (<1 m) increasing with increasing age; a slight reverse was seen for high-level falls although the relationship between age and high-level falls is less clear. Many cases lacked information on intent of injury (i.e., unintentional or intentional) (*N* = 356; 36.6 %) as well as cause due to work-related injuries (*N* = 348; 35.8 %). Among cases with information, 4.3 % (*N* = 25) were due to intentional self-harm and 9.9 % (*N* = 57) were due to a work-related injuries.

**Table 3:** Specific causes of TSCI

	Gender		Age Categories					
Categories	Females	Males	16–30	31–45	46–60	61–75	76+	Total (%)
*Transport-related (22.9 %; N = 213)*								
Bicycle crash	8 %	14 %	3 %	11 %	13 %	40 %	20 %	28 (13 %)
Car crash	47 %	25 %	40 %	22 %	25 %	20 %	20 %	61 (29 %)
Motorcycle	11 %	32 %	33 %	39 %	25 %	13 %	10 %	61 (29 %)
Moped	0 %	6 %	1 %	4 %	8 %	3 %	20 %	10 (5 %)
Pedestrian hit by vehicle	6 %	3 %	1 %	2 %	10 %	0 %	0 %	7 (3 %)
Transport accident, other/unspecified	28 %	20 %	21 %	22 %	19 %	23 %	30 %	46 (22 %)
*Sports- and leisure-related TSCI (26.5 %; N = 248)*								
Swimming/Diving	7 %	16 %	23 %	13 %	8 %	3 %	20 %	36 (15 %)
Hiking	7 %	4 %	2 %	3 %	6 %	10 %	20 %	11 (4 %)
Paragliding/Parachuting	11 %	22 %	14 %	32 %	20 %	7 %	0 %	49 (20 %)
Skiing/Snowboarding	33 %	19 %	26 %	16 %	20 %	28 %	0 %	53 (21 %)
Horseback riding	18 %	2 %	5 %	3 %	10 %	3 %	0 %	12 (5 %)
Mountain biking/bicycling	9 %	11 %	6 %	11 %	12 %	21 %	20 %	27 (11 %)
Motocross	0 %	5 %	7 %	4 %	4 %	0 %	0 %	11 (4 %)
Other	13 %	16 %	13 %	14 %	16 %	21 %	40 %	38 (15 %)
Missing	2 %	5 %	5 %	5 %	2 %	7 %	0 %	11 (4 %)
*Fall-related TSCI (37.1 %; N = 346)*								
Fall from tree/off ladder	5 %	15 %	2 %	16 %	13 %	19 %	7 %	41 (12 %)
Fall from window/balcony/roof	13 %	10 %	28 %	19 %	11 %	0 %	1 %	37 (11 %)
Tripped	17 %	6 %	2 %	1 %	6 %	15 %	21 %	34 (10 %)
Tripped, stairs	11 %	12 %	4 %	4 %	11 %	18 %	17 %	40 (12 %)
Construction/farm-related	3 %	9 %	8 %	12 %	15 %	0 %	3 %	25 (7 %)
Other (Bed, toilet, fall due to health condition, suicide, etc.)	27 %	21 %	25 %	27 %	21 %	24 %	18 %	79 (23 %)
Unclear	24 %	23 %	28 %	18 %	21 %	22 %	29 %	81 (23 %)
Missing	1 %	3 %	4 %	1 %	3 %	1 %	4 %	9 (3 %)

Percentages presented are for the total by column according to each category (i.e., gender and age)

## Discussion

This study found that injury rates for Switzerland varied according to both age and sex with the highest annual IRs observed among men, and the greatest proportion of TSCIs occurring in individuals between 16 and 30 years. The most common cause of TSCI was falls, for which incidence steadily increased with increasing age. In comparison with other European countries, the overall annual IR of TSCI in Switzerland is intermediate . The injury rates reported within this study are the first nationwide estimates for Switzerland that utilize rehabilitation-based data and provide detailed information for prevention and comparison purposes.

### Comparison of Swiss IRs to other countries

In comparison with other European countries, annual IRs for Switzerland were intermediately located; considering only those studies reported that have similar age restrictions, European annual incidence rates ranged between 8.3 and 33.6 per million (Denmark and Greece, respectively) (Jazayeri et al. 2014; Bjornshave Noe et al. 2015). Greece had the highest annual TSCI IR reported in Europe, with 51 % attributed to transport-related injuries, followed by 37 % due to falls; this is in conflict with this study’s findings, which found the majority of TSCIs to be due to falls. This discrepancy could in part be due to traffic safety legislation and compliance. For example, seat belt use in Switzerland was roughly 92 % for drivers and 72 % for rear-seat passengers in 2013, while in Greece it was below 80 % (bfu 2013; ETSC 2011). The lowest annual IR of TSCI was reported for the Denmark, which reported 36.8 % of TSCI cases attributed to transport-related accidents, and 35.5 % due to falls. The low reported annual incidence rate in Denmark could in part be due to the fact that it is a single-center rehabilitation-based study with a regional catchment area, as well as the potential for patients with less severe TSCIs to not be referred to the center from which the study sample arose (Ackery et al. 2004; Bjornshave Noe et al. 2015). Contrary to Greece, in Denmark 94 % of drivers and 81 % of rear seat passengers wore seatbelts (OECD 2014). In similarly high-income countries, in contrast to European incidence rates, annual incidence rates estimated for the United States and Canada were much higher, while Australian estimates were similar (New et al. 2015; Noonan et al. 2012; Jain et al. 2015). Recent annual incidence rate estimates for the United States were 54.0 per million population, 41.0 per million for Canada and 21.0 per million in Australia (based on individuals admitted for rehabilitation and including children admitted to a pediatric trauma hospital) (Noonan et al. 2012; Jain et al. 2015; New et al. 2015). Neither the Canadian nor the Australian study provided information on cause of TSCI. However, there is an evident difference in etiology in the American study, as a large proportion of TSCIs were caused by firearm injuries; an issue that is almost nonexistent in the European setting (Noonan et al. 2012; Jain et al. 2015). Overall differences in reported annual incidence rates might also be due to many studies excluding individuals who died on the way to the hospital, or excluding those who died during acute care. For example, one study by Sabre et al. found that 53 % of patients died before hospitalization in Estonia, equating to more than a two-fold increase in annual incidence of TSCI (Sabre et al. 2014). Another study in Alberta, Canada found that annual incidence rates were nearly 20 % higher when including individuals that died before hospital admission (Dryden et al. 2003). Differences in the etiology and country-specific regulations (e.g., transport-related or drug and alcohol restrictions) of TSCI could also account for discrepancies between reported incidence rates.

### Etiology and prevention

To summarize the implications of the results from this study for prevention efforts and illustrate a concept and it’s use, we will relate the results to a Haddon Matrix approach in the following paragraphs. The Haddon Matrix is a framework used to conceptualize strategies for prevention and response at both the individual- and population-level (Haddon 1968, 1980). Briefly, it is primarily composed of two components: causal factors (*host/person*, *vehicle* through which the host is injured, and the *physical* and *social environment*) and phases of the injury event (i.e., *pre-event*, *event*, and *post-event*) (Runyan 2003). Using these two components in conjunction facilitates the identification of risk factors and countermeasures effective in reducing injury incidence or severity at each phase of the injury event. In the context of this paper, we focus specifically on the *pre-event* and *event phases*.

Within Switzerland, the most common causes of TSCI were falls and sports/leisure-related events; an observation consistent with previous literature (Lee et al. 2014; Cripps et al. 2011). This study observed a trend of increasing incidence of falls with increasing age, and specifically low falls with increasing age. Among fall-related TSCIs in those aged 76 and older, over 80 % were due to low-level falls; for individuals 16 to 30 years old, only about 10 % were due to low-level falls. This pattern is supported by previous literature that has similarly found higher falls to occur mainly in younger age groups, decreasing with increasing age, in contrast to the proportion of low-level falls which increase with age (Feng et al. 2011; Knutsdottir et al. 2012; Chen et al. 2015). Aging is often associated with balance issues, muscle diminishing, frailty, and osteoporosis, which is characterized by the deterioration of skeletal mass and thereby a reduction in supporting structures to withstand a mechanical insult, potentially influencing risk of TSCI in geriatric populations (i.e., the *host/person*) (Pintar et al. 1998; Silver and Einhorn 1995; Madureira et al. 2007; Maki et al. 1994). Occurring most often in older ages, the *vehicles* through which injury occurs are low-level falls due to tripping (same-level fall) or tripping down the stairs, which could be due to declining leg strength necessary for fall prevention (Pijnappels et al. 2008; Stevens et al. 2014). A study by Chen et al. found an increase in falls with increasing age due to tripping on stairs/steps and same-level falls (Chen et al. 2015). As the number of older adults increases, so too does the magnitude of TSCIs due to falls, therefore prevention strategies aimed at the elderly or aging population is imperative. Hinting towards this is a recent study by Bjørnshave Noe et al. which found that over the past twelve years, the median age at injury has increased along with the percentage of falls, with incidence rate ratios (IRRs) for fall-related TSCIs more than two times greater in the last time period (1990–1994) as compared to the first (1990–1994) (adjusted for age and gender) (Bjornshave Noe et al. 2015). Previous research has identified many measures that could be taken during the *pre-event phase* that would reduce the risk of falling among geriatric populations (Kannus et al. 2005). For example, towards the reduction of the proportion of SCIs due to falling in the *pre-event phase*, prevention efforts could focus on strength, balance and resistance training for the *host/person*, the addition of handrails or level seats could modify the *vehicle*, and home hazard assessment and modification such as the removal of loose carpets and clutter, the *physical environment* (Demura et al. 2011; WHO 2013; Robertson et al. 2002). vitamin D supplementation during the *pre-event phase* could in turn modify the risk of injury to the *host/person* during the *event phase*, as it has been found to be helpful in reducing the risk of fall-related fractures and the rate of falls among individuals with reduced levels of vitamin D (Gillespie et al. 2009). Additionally, during the *event phase*, the use of anti-slip shoe devices worn by the *host/person* could help reduce the rate of falls in icy conditions (Gillespie et al. 2009), as well as modifications to the *social environment* such as anti-slip flooring requirements.

The second most-common cause of TSCI in Switzerland was due to sports- and leisure-related activities. Switzerland is a high-income country with access to a diverse landscape including many mountains, and an active population, with roughly 56 % of the population participating in sports-related activities (i.e., sports, gymnastics, or fitness) (Storni et al. 2013). A recent survey found that between 2007 and 2011, 38.2 % of non-occupational incidents were sports-related; winter-related sports accounted for 22.5 % of these injuries (bfu 2014). The large proportion of the Swiss population partaking in sports-related activities (i.e., the *host/person*) reflects the leading cause of TSCIs among younger ages. In this study, the *vehicle* of sports-related TSCIs was most often skiing/snowboarding, with the highest proportion of injuries observed among 16 to 30 year olds. A previous review found TSCI incidence due to skiing/snowboarding accidents between 1 and 13 %, with evidence from a few studies pointing towards increasing rates over time of TSCIs among skiers and snowboarders, likely attributed to advances in ski/snowboard equipment (Ackery et al. 2007). Given the large proportion of sports-related TSCIs in Switzerland attributable to winter activities, especially skiing and snowboarding, *pre-event* prevention efforts could focus on the *host/person* through education and training in safety measures such as the Alpine Responsibility Code (WHO 2013; Ackery et al. 2007).

Evidence in other European and developed countries have shown TSCIs due to traffic-related events have decreased in recent years (Bjornshave Noe et al. 2015; Lee et al. 2014). This can likely be attributed in part to changes of the *physical and social environment* at the level of the *pre-event phase* such as the introduction of speed limits on roads and reassessment of driving ability at older ages. Further, seatbelt requirements related to seatbelt use (*host/person*), presence of seatbelts (*vehicle*), and laws mandating seatbelt use (*social and physical environment*) have reduced the incidence of road traffic injuries in Switzerland and globally at the *event phase* (FEDRO 2014; WHO 2004; Lieutaud et al. 2012). However, the relatively large proportion of transport-related TSCIs, specifically due to car and motorcycle crashes, suggest further scope for prevention efforts.

### Limitations and strengths

A strength of this study is the availability of data according to demographic and lesion characteristics, which made it possible to calculate detailed rates for SCI-specific characteristics, stratified by age and sex; additionally, the large study sample allows for increased precision of estimates. Discordance in reported annual incidence rates could be due to differences in study quality, coverage, and general study characteristics, making it difficult to draw conclusions on the true placement of Swiss annual incidence rates in a worldwide, or even European, context. However, given the quality checks that ensured both accuracy and uniqueness of included records, we believe the SwiSCI medical record data to produce reliable, rehabilitation-based estimates of TSCI in Switzerland and to be of high quality. Although we cannot fully exclude the risk of coding inaccuracies, even after careful screening of medical records, differential misclassification is unlikely, and the conceivably minor extent of non-differential misclassification presumably had a minor impact on reported annual incidence rates (Jurek et al. 2005). An additional strength of this study is that it provides cause-specific incidence rates, which allows for the identification of groups for prevention. Furthermore, although data were not available for all cases of fall-related TSCIs, the similarity of distributions in demographic and SCI-specific characteristics between the sample and the complete data set suggested minimal sampling bias related to the level of falls.

This study included only those cases admitted to a specialized rehabilitation center, all of which are included within the sampling frame of the present study. This implies that those who perish onsite, those who perish during acute care, as well as those who received first rehabilitation in a non-specialized, neurological rehabilitation clinic, would potentially be missed. A future study comparing rehabilitation-based incident cases to incident cases identified using hospital statistics of Switzerland would allow for an estimation of the extent of this underestimation. A study in The Netherlands found that complete lesions were more often discharged to specialized rehabilitation centers than incomplete lesions (78 and 39 %, respectively) (Nijendijk et al. 2014). We therefore similarly expect that the SwiSCI study to reliably captures cases with severe injuries, but acknowledge that it may miss less severe injuries. Furthermore, although there is no reason to suspect broad categories of causes of TSCI to systematically differ in reporting, as there is a large amount of missing information for work-related injuries and intent of injury, these statistics should be interpreted with caution. In Switzerland, intentional injuries forfeit coverage by accident insurance, which could in turn impact reporting, leading to an underestimation of the true proportion of intentional injuries (Le Conseil fédéral 2000). Finally, socioeconomic status or other factors that might impact risk and could impact intervention programs could not be evaluated.

## Conclusions

The estimated annual incidence rates of TSCI for Switzerland are comparable to other European countries and identified several groups at higher risk for TSCI within Switzerland, towards which prevention efforts should be aimed. In line with the need for a population-approach to identify and control causes of incidence (Rose 2001; Doyle et al. 2006), this study underscores the need for prevention efforts to target males; individuals between the ages of 16 to 30 for sports- and leisure-related TSCIs; and fall-related TSCIs among the elderly (76 and older), especially due to tripping. As established by a recent report, discrepancies currently exist in terms of trends in TSCI (WHO 2013), therefore future studies focused on country-specific incidence rates are necessary for identification of effective prevention strategies in various settings. Moving forward, the Haddon Matrix is a valuable tool for the conceptualization and identification of targeted prevention strategies (WHO 2013; Runyan 2003).

## Abbreviations

AIS: American Spinal Injury Association Impairment Scale
ASIA: American Spinal Injury Association
ID: identification
IR: incidence rate
IRR: incidence rate ratio
ISCoS: International Spinal Cord Society
SCI: spinal cord injury
SwiSCI: Swiss Spinal Cord Injury study
TSCI: traumatic spinal cord injury
WHO: World Health Organization
